# Patent Human Infections with the Whipworm, *Trichuris trichiura*, Are Not Associated with Alterations in the Faecal Microbiota

**DOI:** 10.1371/journal.pone.0076573

**Published:** 2013-10-04

**Authors:** Philip Cooper, Alan W. Walker, Jorge Reyes, Martha Chico, Susannah J. Salter, Maritza Vaca, Julian Parkhill

**Affiliations:** 1 Fundación Ecuatoriana Para la Investigación en Salud, Quito, Ecuador; 2 Universidad San Francisco de Quito, Quito, Ecuador; 3 Liverpool School of Tropical Medicine, Liverpool, United Kingdom; 4 Wellcome Trust Sanger Institute, Wellcome Trust Genome Campus, Hinxton, United Kingdom; Charité-University Medicine Berlin, Germany

## Abstract

**Background:**

The soil-transmitted helminth (STH), *Trichuris trichiura* colonises the human large intestine where it may modify inflammatory responses, an effect possibly mediated through alterations in the intestinal microbiota. We hypothesised that patent *T. trichiura* infections would be associated with altered faecal microbiota and that anthelmintic treatment would induce a microbiota resembling more closely that observed in uninfected individuals.

**Materials and Methods:**

School children in Ecuador were screened for STH infections and allocated to 3 groups: uninfected, *T. trichiura* only, and mixed infections with *T. trichiura* and *Ascaris lumbricoides*. A sample of uninfected children and those with *T. trichiura* infections only were given anthelmintic treatment. Bacterial community profiles in faecal samples were studied by 454 pyrosequencing of 16 S rRNA genes.

**Results:**

Microbiota analyses of faeces were done for 97 children: 30 were uninfected, 17 were infected with *T. trichiura*, and 50 with *T. trichiura* and *A. lumbricoides*. Post-treatment samples were analyzed for 14 children initially infected with *T. trichiura* alone and for 21 uninfected children. Treatment resulted in 100% cure of STH infections. Comparisons of the microbiota at different taxonomic levels showed no statistically significant differences in composition between uninfected children and those with *T. trichiura* infections. We observed a decreased proportional abundance of a few bacterial genera from the Clostridia class of Firmicutes and a reduced bacterial diversity among children with mixed infections compared to the other two groups, indicating a possible specific effect of *A. lumbricoides* infection. Anthelmintic treatment of children with *T. trichiura* did not alter faecal microbiota composition.

**Discussion:**

Our data indicate that patent human infections with *T. trichiura* may have no effect on faecal microbiota but that *A. lumbricoides* colonisation might be associated with a disturbed microbiota. Our results also catalogue the microbiota of rural Ecuadorians and indicate differences with individuals from more urban industrialised societies.

## Introduction

Soil-transmitted helminth parasites (STH, also known as geohelminths or intestinal helminths) are estimated to infect 2 billion humans worldwide [1]. The most common STH parasites are the roundworm *Ascaris lumbricoides* and the whipworm *Trichuris trichiura* [1], that are acquired during the second year of life in endemic areas. Adult parasites of *A. lumbricoides* reside in the small intestine while those of *T. trichiura* are found in the caecum. Adult STH parasites may survive for several years and human infections are associated with significant morbidity, particularly through effects on growth and nutrition [1].

*Trichuris* parasites are considered to have potent immune regulatory effects within the host both locally in the colon (therapeutic infections with pig whipworm, *T. suis*, have been associated with an improvement in symptoms of inflammatory bowel disease [2],[3]) but also distally, having been associated with protection against allergy [4],[5]. Dampening of inflammatory responses in the intestine could be an important survival strategy. Asymptomatic chronic infections are associated with very mild histological alterations that are indistinguishable from local uninfected controls [6]. The mechanisms by which *T. trichiura* may mediate immune regulatory effects are not well understood. One mechanism is through the induction of immune regulatory cytokines such as IL-10, which are increased during chronic infections in humans [7],[8]. An alternative effect might be through alterations in the intestinal microbiota, which plays several key roles in the development, maintenance and regulation of host immunity [9],[10]. Recent studies of the intestinal microbiota in mice infected with *T. muris*, in pigs infected with *T. suis*, and in rhesus macaques infected with *T. trichiura* have provided evidence that the presence of *Trichuris* parasites is associated with an altered microbiota [11],[12], [13],[14],[15]].

In the present study we tested the hypothesis that STH infections of humans affect the composition of faecal microbiota and that anthelmintic treatment would revert this altered microbiota composition towards that observed in uninfected controls. We examined the effects of *T. trichiura* infections on faecal microbiota by comparison with local controls and also evaluated the effects of a curative course of anthelmintic treatment on composition of the intestinal microbiota. We chose *T. trichiura* as the model STH infection because of its immune regulatory effects and because it is located in the large intestine and thus any effects might be more readily detected by faecal sampling. We also evaluated the effects of mixed infections with *A. lumbricoides* and *T. trichiura* on the intestinal microbiota.

## Materials and Methods

### Study population, sample and data collection

**Figure 1:**
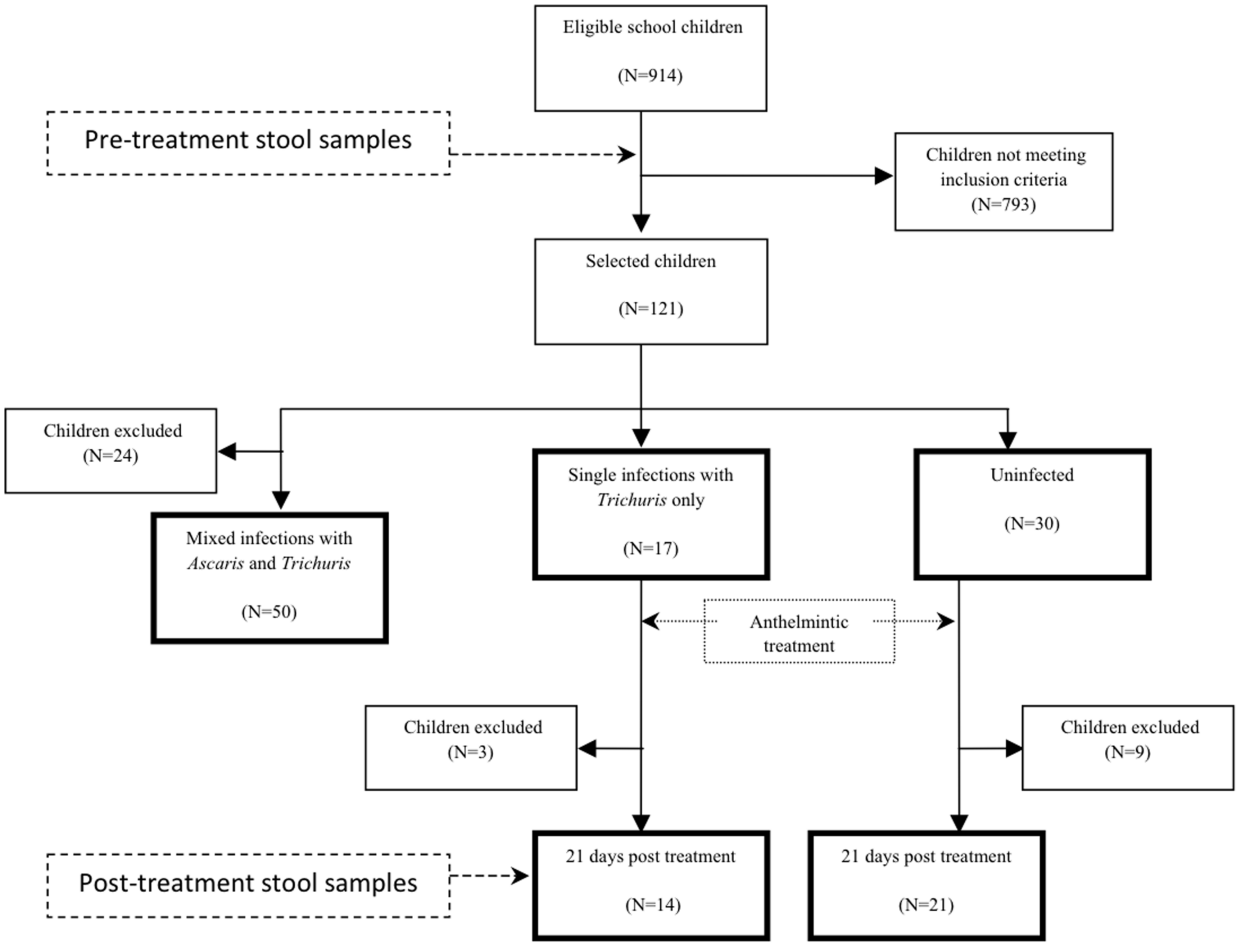
Flow diagram illustrating selection of study subjects for analysis. Boxes in bold represent the children included in the present analysis.

The fieldwork for this observational study was done between June and September 2009. We evaluated for inclusion a total of 914 children attending 3 rural villages in the District Eloy Alfaro, Esmeraldas Province, Ecuador, where we had previously observed a high prevalence of STH infection [16]. Two to three stool samples were collected over the period of a month from all children and were examined for the presence of STH eggs and larvae. To investigate the effects of STH infections and anthelmintic treatment on intestinal microbiota we classified children into 3 groups according to the results of stool examinations: Uninfected controls - no STH parasites detected in any of a minimum of 3 stool samples; *T. trichiura* infection only – presence of *T. trichiura* but no other STH parasite in all stool samples [specific effects of *T. trichiura* on microbiota]; mixed infections - presence of *A. lumbricoides* and *T. trichiura* in all stool samples [effects of mixed STH infections on microbiota]. Children with inconsistent findings between stool samples were excluded. We selected for further evaluation a total of 121 of these children who met the study inclusion criteria: belonging to one of the 3 infection groups as described above, aged 8 to 14 years, had taken neither antibiotics in the previous month nor anthelmintic treatment in the previous 3 months before the start of the study, and were afebrile and asymptomatic at the time of sampling. Figure 1 provides a flow diagram showing the selection of study children. To evaluate the effects of anthelmintic treatment on intestinal microbiota, all 17 children in the *T. trichiura*-only infection group and 21 of the 30 children in the Uninfected group received albendazole 400 mg twice daily for 3 days and a single dose of 200 µg/kg of ivermectin. The treatment regimen was designed to ensure complete cure of all STH infections: albendazole is optimal for the treatment of ascariasis and ivermectin for the treatment of strongyloidiasis [17], and a combination of the two is optimal for the treatment of trichuriasis [18]. Both drugs are extremely safe at the doses used [18],[19]. The treatment protocol was designed by PJC and all treatments were directly observed by JR and MV. Single stool samples were collected from each child at 7 and 21 days following treatment. Sampling at 7 and 21 days was chosen to document cure of STH infections and 21 days for measurement of faecal microbiota. A 21-day time point was chosen to determine the short-term effects on microbiota of the elimination of STH parasites and was a compromise between allowing time for microbiota to recover following parasite expulsion and avoiding possible interference by new pre-patent infections becoming established in the intestine given that the study children continued to reside in an endemic environment. At the end of the study all untreated children were treated with the same anthelmintic treatment regimen (i.e. within 3 months of the detection of infections). All children were healthy and participation in the study did not change their clinical treatment. All stool samples were examined using the modified Kato-Katz method [20] for the identification and quantification of STH eggs and two slides were read for each sample. A 500 mg aliquot of stool was placed in a 2 ml microtube and an equal volume of 90% ethanol was added. The sample was mixed vigorously with a vortex for 1 minute and then frozen and stored at −20°C until extraction of genomic DNA.

### DNA Extraction

Total DNA was isolated from 250 mg of faeces using the FastDNA® SPIN Kit for Soil (MP Biochemicals, Irvine, CA) in conjunction with a FastPrep-24 Instrument (MP Biochemicals) following the manufacturers instructions. Extracted DNA was re-suspended in 50 µl of pyrogen-free water and stored at −20°C. DNA samples were shipped to the Wellcome Trust Sanger Institute on dry ice for PCR and 454 pyrosequencing.

### Amplification of 16 S rRNA Genes

Polymerase chain reaction (PCR) was used to amplify variable regions 3 to 5 (V3-V5) of the 16 S rRNA gene. Samples were PCRed using primers 454B_338F (5′-CCTATCCCCTGTGTGCCTTGGCAGTCTCAGACTCCTACGGGAGGCAGCAG-3′) and 454A_926R (5′-CCATCTCATCCCTGCGTGTCTCCGACTCAG-barcode-CCGTCAATTCMTTTRAGT-3′) containing sample specific barcodes and Roche 454 Lib-L adaptor sequences (shown in bold in primer sequences above). See Table S1 for full barcode and primer sequences. To minimise PCR nucleotide insertion mistakes, a high fidelity Taq polymerase (AccuPrime™, Invitrogen, Carlsbad, USA) was used, and samples were amplified in quadruplicate reactions with 20 cycles each and then pooled. A mastermix for sequencing was created by pooling together roughly equimolar amounts of each sample, as measured by a Qubit fluorometer (Invitrogen, Carlsbad, USA).

### Pyrosequencing and Data Analysis

The DNA amplicons were pyrosequenced using a GS FLX Titanium 454 (Roche Diagnostics, Oakland) machine following the manufacturer's Lib-L kit protocols and were initially processed using the “Amplicon” configuration of the 454 Sequencing System software. This sequence data is available in the European Nucleotide Archive (ENA) short-read archive under the sample accession number ERS236495 and/or the study accession number ERP002465. Downstream data analysis was performed using the mothur software package [21]. Briefly, reads were filtered for quality by truncating them once average quality scores dropped below 35 across a rolling window of 50 bases. Following this step all reads less than 350 bases in length, those with any mismatches to the barcode or 16 S rRNA gene primer sequence, and those with any ambiguous bases or with homopolymeric stretches of longer than 8 bases were discarded. Chimeras were removed using Perseus software [22], as implemented in mothur. Following these data cleaning steps a median of 2286 sequences remained per sample. Sequences were aligned to the reference SILVA database provided in mothur, then clustered into Operational Taxonomic Units (OTUs) at 97% sequence identity using the default average neighbour setting. Phylogenetic classifications were assigned to each OTU at all taxonomic levels from Phylum to Genus using the reference Ribosomal Database Project database (RDP) provided in mothur. Classifications for selected OTUs, typically the most abundant ones, were further verified by checking similarities using MegaBLAST against the NCBI nucleotide archive [23]. Shannon diversity index scores were calculated using mothur [21], after samples were randomly subsampled to a depth of 500 sequence reads per sample to ensure that final diversity scores were not influenced by differential sequencing depths.

### Statistical analysis

Categorical variables by group were compared using the chi-squared test and continuous variables using the Mann Whitney test. Paired analyses within groups were done using the Wilcoxon matched-pairs signed-ranks test. The Bonferroni correction was used for multiple comparisons. The primary analysis for this study was to evaluate the effect of single infections with *T. trichiura* on faecal microbiota – we did this by comparing microbiota composition between children infected with *T. trichiura* only and uninfected children and then by looking at the effect of anthelmintic treatment among children with single *T. trichiura* infections. The effect of anthelmintic treatment *per se* was evaluated by comparing paired samples from uninfected children before and after treatment. The effect of mixed infections on microbiota composition was evaluated by comparing samples from children with mixed infections with those from uninfected children.

### Ethics statement

The study protocol was approved by the Institutional Review Board of the Universidad San Francisco de Quito, Quito, Ecuador. Written informed consent was obtained from the parent of each child and signed minor assent from the child.

## Results

### Characteristics of study population

We analysed samples from a total of 97 children from the three infection groups (uninfected, 30; *T. trichiura* only, 17; and mixed infections with *A. lumbricoides* and *T. trichiura*, 50). Demographic, socioeconomic, STH infection, and other relevant characteristics for the three study groups are provided in Table 1. Most of these variables did not differ significantly across groups. Children with STH infections (*T. trichiura* only and mixed groups) were more likely to defecate in the open (P<0.001), have a lower monthly household income (P = 0.02), and less likely to have received anthelmintic treatment in the previous six months (P = 0.03) compared to uninfected children. Infected children were also more likely to have more poorly educated mothers, although this was not statistically significant (P = 0.08). All children with single *T. trichiura* infections and 21 of the 30 uninfected children received anthelmintic treatment. Following treatment none of the children had evidence of any STH infection at 7 and 21 days following treatment, indicating that the treatment regimen cured all *T. trichiura* infections. Although we did not collect dietary information from this study cohort, a parentally-administered survey conducted in two of the three study communities for children aged 8–14 years showed a diet rich in fibre in which unprocessed rice and plantain were consumed daily by almost all the individuals (see Table S2).

**Table 1:** Characteristics of study population stratified by infection status with soil-transmitted helminth infections. Epg  =  eggs per gram.

Variable	Uninfected	*T. trichiura* only	Mixed infections
	(N = 30)	(N = 17)	(N = 50)
Age (years)			
Mean (range)	11 (8–14)	10 (8–14)	10 (8–14)
Gender			
Male/Female	15/15	8-Sep	27/23
Monthly income			
Mean US$ (range)	238 (15–1000)	161 (60–300)	140 (40–300)
Maternal educational level (%)			
Illiterate	28	35	55
Completed primary	45	47	37
Completed secondary	27	18	8
Crowding (person/room)			
Mean (range)	3 (1–7)	4 (1–10)	4 (1–10)
Bathroom			
WC	48	0	0
Latrine	24	6	28
Outside	28	94	72
Treatment in the last 6 months			
Yes (%)	59	35	28
STH infections			
*A. lumbricoides*			
Prevalence (%)	0	0	100
Intensity (median epg [range])	0	0	32,713 (467–336,887)
*T. trichiura*			
Prevalence (%)	0	100	100
Intensity (median epg [range])	0	2,893 (47–23,913)	5,402 (23–76,836)

### General characteristics of the faecal microbiota in Ecuadorian children

We examined the faecal microbiota in a total of 132 stool samples from 97 children living in rural Ecuador - post-treatment samples were collected from 35 of the 97 children as shown in Figure 1. We obtained 999,796 raw sequences from all samples and after strict filtering for quality and removal of chimeric sequences a total of 306,354 sequences remained, which were clustered into 1,106 distinct OTUs at a 97% sequence identity level (see Tables S3 and S4 for a detailed description of each OTU). In common with other 16 S rRNA gene-based surveys of the human intestinal microbiota, our analysis revealed that the vast majority of the sequences belonged to the Firmicutes (67.4%) and Bacteroidetes (21.2%) phyla. More in depth analysis at finer taxonomic levels revealed further commonalities with previously published microbiota analyses, as well as some intriguing differences.

As is also typical with individuals from Western/industrialised countries the majority of species within the Firmicutes phylum belonged to the *Lachnospiraceae* (formerly clostridial cluster XIV) and *Ruminococcaceae* (formerly clostridial cluster IV) families. Furthermore, the most abundant organism in our Ecuadorian dataset was the Firmicutes species *Faecalibacterium prausnitzii* (16.6% of total sequences recovered), which has previously been reported to be one of the most abundant organisms in individuals from the Western/industrialised world [24] and has also received much attention recently as a potentially anti-inflammatory species [25].

**Figure 2:**
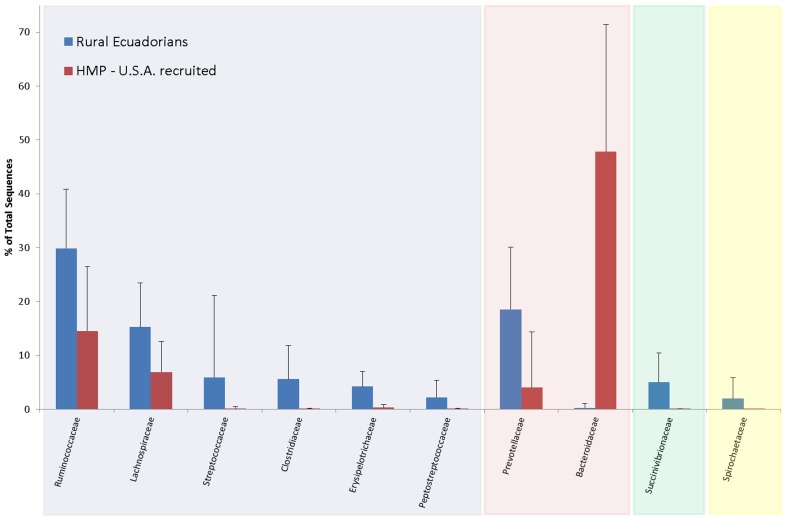
Mean proportional abundance of selected bacterial taxonomic Families in 97 rural Ecuadorian children using pre-treatment samples, compared with U.S.-recruited adults. Low abundance taxa were excluded from this figure for the sake of clarity. Families with blue background belong to the Firmicutes phylum, red  =  Bacteroidetes, green  =  Proteobacteria, yellow  =  Spirochaetes (mostly *Treponema* spp.). U.S. data generated by the Human Microbiome Project [43]. Errors bars show standard deviation from the mean.

Despite these broad similarities there was also some evidence for the development of distinct microbiota structures in the rural Ecuadorian population sampled for the current study. For example, the fourth most abundant OTU was most similar to a member of the *Succinivibrio* genus of the gamma-proteobacteria (median abundance of 3.1%, interquartile range 0.4% to 8.2%, maximum of 27.4%) (Table S3). While similar organisms are common inhabitants of rumens [26] to our knowledge they have never been observed at such abundant proportions in human faecal samples from Western subjects. Similarly, an OTU related to *Sarcina ventriculi*, an organism that is very rarely recovered from Western individuals but has previously been identified as common in developing countries [27], was detected in 59.8% of the faecal samples provided by our rural Ecuadorian cohort. We also detected a relatively high proportional load of *Treponema* spp. (median abundance of 0.2%, interquartile range 0 to 2.3%, maximum of 25.9%) in the Ecuadorian cohort. Finally, when analysing sequences belonging to the Bacteroidetes phylum in more depth we found that the vast majority belonged to the *Prevotella* genus (median abundance of 16.9%, interquartile range 8.5% to 25.6%, maximum of 49.3%), with only a very small proportion belonging to the *Bacteroides* genus (median abundance of 0.1%, interquartile range 0 to 0.2%, maximum of 8.9%), which, in contrast, is typically one of the most abundant genera in Western subjects [28],[29]. The most abundant bacterial Families present in Ecuadorian samples, and their comparative proportional abundances in the Human Microbiome Project's cohort of US-recruited individuals, are shown in Figure 2.

Given the potential for pathogenic microbes to alter host immune responses and microbiota profiles we also searched the OTU list for the presence of overtly pathogenic bacteria. We observed a very small number of sequences matching *Campylobacter jejuni* in three samples (two individuals with both *T. trichiura* and *A. lumbricoides* infection and one individual free from helminth infection following treatment with anthelmintics). The significance of this observation is unclear although concurrent trichuriasis has been associated with severe *C. jejuni*-associated colitis in humans [30] and pigs [31] in previous studies. We did not detect any other “classic” overt bacterial pathogens such as *Salmonella* spp., *Yersinia enterocolitica*, *Vibrio cholerae, Staphylococcus aureus*, and *Aeromonas hydrophila*. Because it is not possible to separate *Shigella* spp. and pathogenic *Escherichia coli* species from commensal *E. coli* strains using just short fragments of the 16 S rRNA gene we could not determine the presence or absence of these potential pathogens. This was also the case with both *Bacillus cereus* and *Clostridium perfringens*, which could not be discriminated from other closely related but non-enteropathogenic species.

### Effects of single infections with *T. trichiura* on faecal microbiota

**Figure 3:**
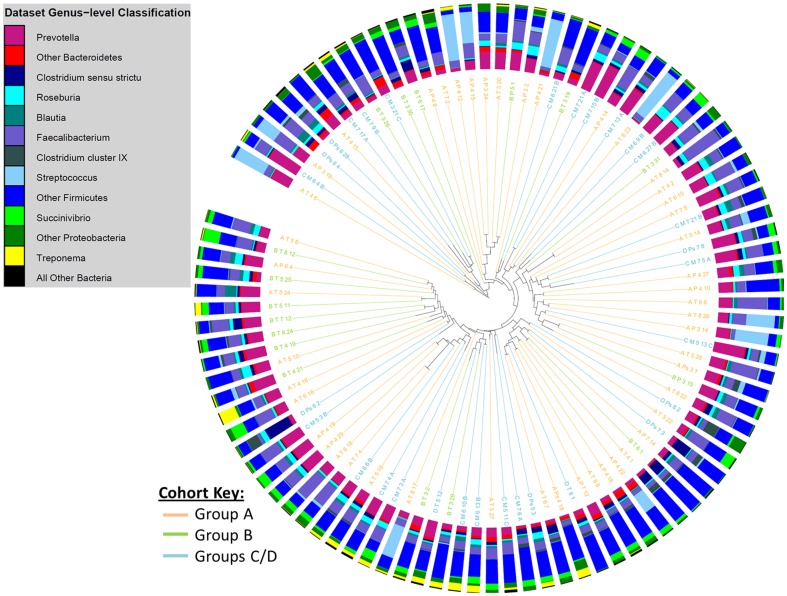
Cluster dendrogram, generated with the Jaccard calculator in the mothur software package, showing similarity in community membership at the OTU-level between faecal samples from different study groups. Surrounding bar charts show the microbiota composition at the genus level for each sample. Group A  =  children infected with mixed infections with *A. lumbricoides* and *T. trichiura* before anthelmintic treatment [N = 50], B  =  children infected with *T. trichiura* only before treatment [N = 17], C/D  =  uninfected children before treatment [N = 30].

**Figure 4:**
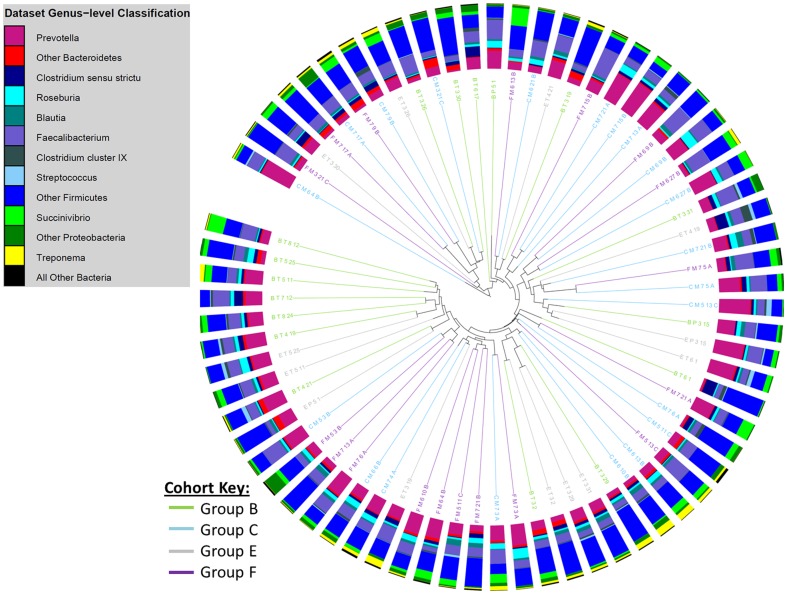
Cluster dendrogram, generated with the Jaccard calculator in the mothur software package, showing similarity in community membership at the OTU-level between faecal samples following anthelmintic treatment. Surrounding bar charts show the microbiota composition at the genus level for each sample. Group B  =  children infected with *T. trichiura* only before treatment [N = 17], E =  children infected with *T. trichiura* only, sampled 21 days post treatment [N = 14], C  =  uninfected children before treatment [N = 21], F  =  uninfected children from Group C, sampled 21 days post treatment [N = 21].

**Figure 5:**
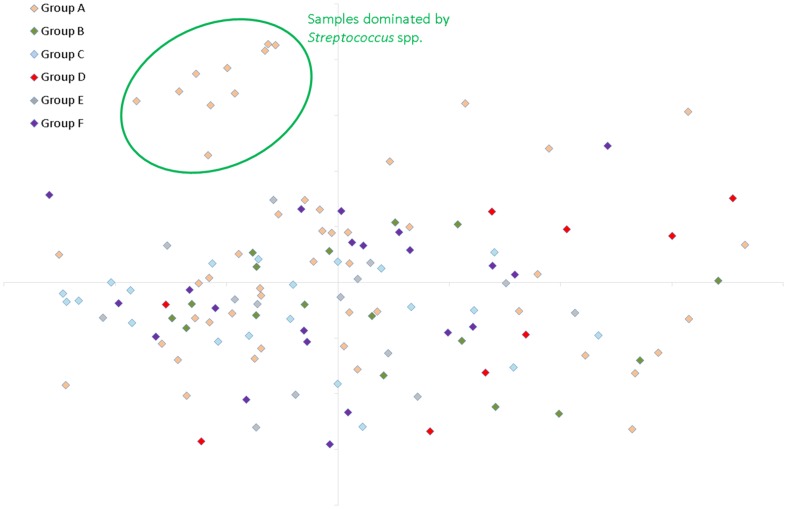
Non-metric multidimensional scaling plot, generated in mothur using the Yue & Clayton theta similarity co-efficient, showing overlap in community structure (including proportional abundance of each OTU) between each of the study groups. Group A  =  children infected with mixed infections with *A. lumbricoides* and *T. trichiura* before anthelmintic treatment [N = 50], B  =  children infected with *T. trichiura* only before treatment [N = 17], C  =  uninfected children before treatment [N = 21], D  =  uninfected children before treatment [N = 9], E  =  children infected with *T. trichiura* only, sampled 21 days post treatment [N = 14], F  =  uninfected children from Group C, sampled 21 days post treatment [N = 21].

The relative abundance of bacterial genera present in faecal samples from children with single *T. trichiura* infections and from uninfected children is shown in Table 2. There were no significant differences between the two groups in the proportional abundances of the bacterial genera identified. Cluster dendrogram and non-metric multidimensional scaling (NMDS) analyses confirmed there was no distinctive separation between the study groups that were infected with *T. trichiura* only and those that were free from STH infection (Figures 3, 4 and 5). We used two calculators to determine the level of dissimilarities between bacterial communities. The Jaccard calculator ignores relative abundance of each OTU and instead examines the level of overlap in community membership by simply observing presence or absence of each OTU across all of the samples. This analysis showed that there was no distinctive clustering of samples based on whether the children were infected with *T. trichiura* alone, infected with *T. trichiura* and *A. lumbricoides* or infected with neither (Figure 3). In addition, Figure 4 shows that, although longitudinally-sampled pairs from the same individual often clustered together (e.g. pre- and post-treatment samples from uninfected subjects represented by groups C and F, respectively) there was no overall clustering of samples based on presence or absence of *T. trichiura*. Similarly, the Theta Yue and Clayton calculator, which does take into account the proportional abundance of each OTU when comparing dissimilarity in community structures, did not show a clear separation of microbiota profiles based on *T. trichiura* infection (Figure 5).

**Table 2:** Relative composition of faecal microbiota by bacterial genus in children with no STH infection (uninfected), children infected with only *T. trichiura*, and those infected with both *T. trichiura* and *A. lumbricoides* (mixed infection).

Genus	Uninfected [C/D] [N = 30]	*Trichuris* only [B] [N = 17]	Mixed infection [A] [N = 50]	P value for C/D vs.B	P value for C/D vs. A	P value for B vs. A
Prevotella	15.7 (6.9–32.1)	15.2 (7.0–27.0)	16.0 (6.8–21.6)	0.60	0.35	0.77
Other Bacteroidetes	2.8 (1.5–4.4)	3.1 (2.1–6.3)	2.3 (1.1–4.2)	0.54	0.38	0.22
Clostridium *sensu stricto*	4.1 (1.9–6.5)	3.7 (2.6–6.7)	1.5 (0.6–4.9)	0.84	0.001 (0.013)	0.004 (0.047)
Roseburia	4.2 (2.8–8.4)	4.2 (2.0–5.3)	2.6 (1.5–5.8)	0.40	0.03	0.41
Blautia	2.6 (1.7–4.0)	2.3 (1.7–3.0)	2.3 (1.3–5.0)	0.30	0.62	0.56
Faecalibacterium	12.9 (10.7–18.5)	16.0 (10.8–22.8)	14.2 (9.4–22.6)	0.48	0.67	0.75
“Clostridium” cluster IX	2.3 (0.5–5.6)	1.7 (1.2–3.1)	0.6 (0.3–1.5)	0.89	0.002 (0.026)	0.002 (0.025)
Streptococcus	0.3 (0.1–1.0)	0.8 (0.3–1.5)	0.9 (0.3–6.8)	0.11	0.007	0.42
Other Firmicutes	29.2 (18.6–38.6)	28.5 (23.3–44.7)	27.8 (17.4–37.5)	0.27	0.76	0.18
Succinivibrio	2.7 (0.7–6.8)	2.7 (0.3–7.6)	2.0 (0.1–8.1)	0.98	0.68	0.63
Other Proteobacteria	3.0 (1.3–6.3)	3.6 (2.3–6.0)	3.4 (1.3–5.0)	0.61	0.97	0.67
Treponema	0.4 (0–3.9)	0.9 (0–2.2)	0 (0–0.5)	0.96	0.005	0.01
All Other Bacteria	0.5 (0.3–1.1)	0.7 (0.4–1.4)	0.4 (0.1–0.9)	0.51	0.07	0.03

Shown are median values and interquartile ranges (brackets).

### Effects of anthelmintic treatment on faecal microbiota in children with single infections with *T. trichiura*

The proportions of OTUs belonging to different bacterial genera among children infected with single *T. trichiura* infections did not alter significantly after treatment (Table 3). Further, there was no effect of anthelmintic treatment *per se* on faecal microbiota composition by comparison of microbiota before and after treatment among uninfected children that were given the same treatment regimen (Table 3) and among all children that received anthelmintic treatment (all matched pairs in Table 3). Cluster dendrograms and NMDS plots, with both the Jaccard and Yue and Clayton calculators confirmed that there was no distinctive separation of samples by anthelmintic treatment (Figures 4 and 5).

**Table 3:** Relative composition of faecal microbiota by bacterial genus before and after treatment for children infected with *T. trichiura* only, uninfected children, and all children that received anthelmintic treatment (all matched pairs).

Genus	*Trichuris* infection only		Uninfected		All matched pairs		P values		
	(N = 14)		(N = 21)		(N = 35)				
	Before (B)	After (E)	Before (C)	After (F)	Before (All)	After (All)	B vs. E	C vs. F	All pairs
Prevotella	14.4 (6.7–27.5)	20.3 (16.0–25.6)	23.3 (11.8–36.7)	12.8 (8.8–21.8)	21.4 (8.5–32.0)	16.2 (11.4–25.6)	0.19	0.04	0.3
Other Bacteroidetes	3.8 (2.2–7.4)	3.0 (2.2–4.0)	2.6 (1.5–3.5)	3.3 (1.1–4.2)	2.9 (1.6–4.6)	3.3 (2.0–3.7)	0.33	0.39	0.87
Clostridium *sensu stricto*	3.6 (1.9–6.7)	4.5 (2.4–7.5)	3.5 (1.6–5.8)	1.4 (1.1–4.2)	3.6 (1.6–6.0)	3.2 (1.3–5.3)	0.64	0.15	0.45
Roseburia	4.4 (2.0–5.7)	2.9 (2.4–4.2)	4.2 (2.4–8.4)	2.5 (1.6–4.9)	4.3 (2.2–8.3)	2.8 (1.9–4.5)	0.47	0.1	0.07
Blautia	2.2 (1.7–3.0)	3.6 (1.5–5.4)	2.8 (1.9–3.9)	4.0 (2.7–5.6)	2.5 (1.8–3.3)	4.0 (2.1–5.6)	0.16	0.18	0.06
Faecalibacterium	14.3 (8.2–25.5)	17.2 (10.1–21.6)	15.3 (11.2–19.3)	15.5 (14.0–26.8)	14.3 (10.9–21.7)	16.4 (11.8–23.2)	0.27	0.17	0.08
“Clostridium” cluster IX	1.7 (0.9–2.6)	1.8 (1.0–2.7)	0.7 (0.4–3.1)	0.7 (0.2–1.5)	1.4 (0.5–3.1)	1.1 (0.5–1.9)	0.51	0.09	0.33
Streptococcus	1.0 (0.6–2.1)	0.9 (0.1–2.5)	0.1 (0–0.6)	0.2 (0–0.5)	0.5 (0.1–1.2)	0.3 (0–1.2)	0.88	0.97	0.9
Other Firmicutes	34.7 (23.3–53.3)	26.4 (22.7–41.9)	28.4 (18.6–35.4)	31.8 (25.1–37.2)	29.9 (18.6–43.4)	31.0 (24.2–41.9)	0.27	0.12	0.71
Succinivibrio	1.7 (0.3–6.9)	1.5 (0.5–7.9)	2.9 (1.5–6.8)	5.1 (1.0–10.6)	2.7 (0.9–6.9)	3.9 (0.6–8.9)	0.59	0.19	0.2
Other Proteobacteria	3.7 (2.3–6.0)	2.3 (1.2–3.5)	2.9 (1.9–5.8)	3.3 (1.5–4.9)	3.1 (1.9–6.0)	2.6 (1.2–4.4)	0.06	0.54	0.1
Treponema	0.7 (0–3.2)	0.5 (0–3.5)	0.6 (0.1–3.9)	0.9 (0.3–2.4)	0.6 (0.1–3.9)	0.9 (0–3.2)	0.95	0.86	0.9
All Other Bacteria	0.6 (0.4–1.4)	0.4 (0.2–1.2)	0.5 (0.3–1.0)	0.7 (0.2–1.4)	0.5 (0.3–1.3)	0.5 (0.2–1.4)	0.33	0.34	0.77

Shown are median values and interquartile ranges (brackets). P values represent comparison of matched pairs pre- and post-treatment.

### Effects of mixed infections with *A. lumbricoides* and *T. trichiura* on faecal microbiota

**Figure 6:**
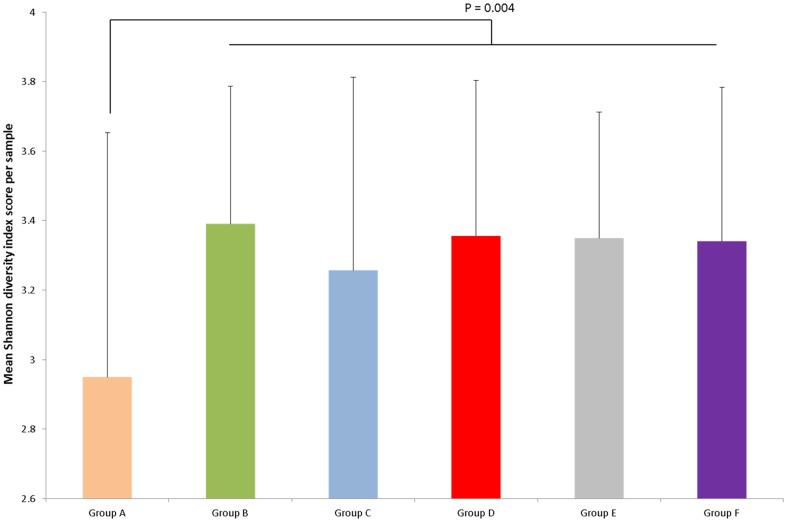
Average Shannon diversity index scores for each of the study groups within the overall Ecuadorian cohort. Group A  =  children infected with mixed infections with *A. lumbricoides* and *T. trichiura* before anthelmintic treatment [N = 50], B  =  children infected with *T. trichiura* only before treatment [N = 17], C  =  uninfected children before treatment [N = 21], D  =  uninfected children before treatment [N = 9], E =  children infected with *T. trichiura* only, sampled 21 days post treatment [N = 14], F  =  uninfected children from Group C, sampled 21 days post treatment [N = 21].

Comparison of faecal microbiota between children with mixed infections and uninfected children showed a significantly greater proportional abundance of OTUs in faecal samples from uninfected children belonged to the *Clostridium sensu stricto* genus (uninfected 4.1% vs. mixed infections 1.5%; P = 0.013, adjusted for multiple comparisons) and uncharacterised clostridial cluster IX bacteria (uninfected 2.3% vs. mixed 0.6%; adjusted P = 0.026) (Table 2). This effect seemed to be attributable to *A. lumbricoides* infection because comparison of these two bacterial genera showed significant differences also between children with mixed infections and those infected with *T. trichiura* only (*Clostridium sensu stricto*, mixed 1.5% vs. *T. trichiura* only 3.7%; adj. P = 0.047: Clostridial cluster IX, mixed 0.6% vs. *T. trichiura* only 1.7%; adj. P = 0.025). A further difference was that overall diversity, as measured using the Shannon diversity index, which takes into account both the number and relative evenness of the OTUs in a given sample for calculating a diversity score, was statistically significantly lower in faecal samples from individuals with mixed infections compared to all of the other samples analysed (P = 0.004) (Figure 6) or compared to all other pre-treatment samples (P = 0.022). Overall there was not a definitive, distinguishing profile associated with mixed infections (Figures 3 and 5), although a subgroup of 10 of the 50 mixed infection samples appeared to be dominated by unusually high proportional abundances of *Streptococcus* spp. (Figure 5). Streptococci are not typically dominant in health, indicating that the microbiota was particularly disturbed in these individuals. Taken together these results indicate that mixed infection, or infection with *A. lumbricoides*, could potentially drive the development of an altered faecal microbiota profile, with reduced proportional abundances of some members of the Clostridia class, an increase in streptococci (in some individuals) and reduced overall diversity.

## Discussion

In the present study we tested the hypothesis that *T. trichiura* mediates its immune modulatory effects through the alteration of the intestinal microbiota, resulting in an increased frequency of bacteria that favour the regulation of inflammation at mucosal sites. However, we observed no effect of single infections with *T. trichiura* on the relative composition of microbiota from faecal samples, and curative treatment of these infections had no effect on faecal microbiota composition in the short-term (i.e. at 3 weeks after treatment). We chose *T. trichiura* as the model infection to measure the effects of STH infections on intestinal microbiota because this parasite has been shown to affect immune regulation in humans in previous studies and it resides in the large intestine.

*Trichuris* spp. are natural parasites of a wide variety of different mammalian hosts [32]. Evidence from previous studies indicates that *Trichuris* infections of animals may modify the intestinal microbiota. Experimental infections with *Trichuris suis* in 7 pigs showed a disturbed microbiota in the proximal colon at 21 [12] and 53 [14] weeks following infection that was associated with changes in the abundance of approximately 13% of bacterial genera and particularly with declines in the relative abundance of *Fibrobacter* and *Ruminococcus*, although these were not reflected in changes in diversity indices at the genus level [12]. Rhesus macaques with idiopathic chronic diarrhoea, an inflammatory bowel disease of monkeys, were infected with *T. trichiura* ova and although patent infections were not established, there was some evidence of clinical improvement in four out of the five monkeys investigated [15]: *T. trichiura* infection was associated with a reduction in bacterial attachment to the colonic mucosa 14 weeks after infection but an increase in bacterial diversity with an increase in the abundance of Tenericutes and Bacteroidetes [15]. Such apparent beneficial effects of *T. trichiura* on diarrhoea could have been mediated through immune mechanisms rather than through changes in microbiota, with the latter being a consequence of the resolution of diarrhoea rather than a direct effect of the parasites.

*Trichuris* species are not the only intestinal helminth parasites that have been associated with alterations in host intestinal microbiota. Infections with *Heligmosomoides polygyrus*, which lives in the duodenum of mice, have been associated with changes in the microbiota of the ileum, characterised by a significant increase in the abundance of the family *Lactobacillaceae,* although no changes were observed in the caecum [33]. In contrast, a study of the abomasal microbiota of cows showed no difference in microbiota between partially immune animals challenged with *Ostertagia ostertagi* compared with controls [11].

Although we did not observe *T. trichiura*-driven effects on the faecal microbiota our results broaden our knowledge in more fundamental ways. The vast majority of microbiota studies have involved sampling of individuals from Western/industrialised societies. Studies involving more rural populations, or individuals from other geographic locations are sorely needed. Our results widen sampling to children living in rural Ecuador, and indicate that there are distinctive microbial signatures associated with these individuals. Of particular interest are the predominance of *Prevotella* spp. at the expense of *Bacteroides* spp. Recent evidence suggests that these two genera are inversely correlated [34],[35], and that *Prevotella* spp. are particularly abundant in rural African populations consuming a high fibre/resistant starch diet [35],[36]. The elevated proportional abundance of *Prevotella* spp., and concurrently low proportional abundances of *Bacteroides* spp. in our study population suggests similar patterns in rural Ecuadorian children. Recent work by Wu *et al* [34] indicates that *Bacteroides* predominance is linked to diets high in saturated fats and animal protein, while *Prevotella* predominance is linked to diets high in carbohydrates and simple sugars [34]. While we were unable to obtain full dietary information from the study subjects, a dietary frequency questionnaire conducted in children of the same age range from two of the three study communities showed a diet in which levels of starch and fibre were high (daily consumption of unprocessed rice and plantain in >90% of children). The presence of other known starch/fibre fermenters such as *Succinovibrio* species and *Sarcina ventriculi*, which is more commonly found in the guts of vegetarians [27], in these Ecuadorian children also supports the hypothesis that diet is a key driver of their microbiota development.

Our study is subject to several important limitations that should be considered in interpreting the study findings. The microbiota of human faeces differs to that of colonic mucosa [37],[38]. As a result, the faecal samples collected in this study do not necessarily represent the microbiota of the caecal mucosa, the principal site of colonisation of *T trichiura*. It is quite possible that the effects on intestinal microbiota of *T. trichiura* are localised to the sites of parasite colonisation. However, due to the rural location and lack of appropriate clinical facilities it was not feasible to collect intestinal biopsy specimens from the children and therefore confirm or refute this hypothesis. A study of the effects of *H. polygyrus* infection on intestinal microbiota showed that differences between infected and uninfected animals could be detected in the terminal ileum but not in the caecum [33].

We selected 21 days as the post-treatment sampling time because we felt this was sufficient time to allow the parasite-altered microbiota to revert to the uninfected state and to minimise the possibility of a new infection. We do not believe that early but transient changes (i.e. immediately after parasite expulsion) that we would have missed are likely to be of biological relevance. However, it is possible that we missed delayed effects on faecal microbiota that might have been detected at a later sampling point. A recent study of the effects of *T. suis* on the porcine colonic microbiota showed that while similar effects were measurable at 21 and 53 days after infection [12], [14], the same effects were observed in infection-challenged but parasite-free pigs at 53 days after infection (i.e. pigs that had expelled their parasites) [14], indicating that *T. suis* could have persistent effects on the colonic microbiota. Our study children were likely to have chronic infections with *T. trichiura* and may have harboured these parasites for years – under such circumstances the microbiota may become permanently altered and not be affected by anthelmintic treatment. Similarly, in an environment of intense transmission with STH parasites, as was the case in the present study, we cannot exclude the possibility that uninfected individuals had been infected with *T. trichiura* infections in the past but had self-cured or treated their infections. The effects of such exposures could be to make the microbiota of uninfected and infected children more similar.

Our inability to detect an effect of *T. trichiura* on faecal microbiota could also be explained by infection intensity. An effect on faecal microbiota of *T. trichiura* might be most clearly demonstrable among children with the highest infection intensities. However, among the 17 children with single *T. trichiura* infections, only 2 (12%) had heavy parasite burdens (≥10,000 eggs per gram [epg] of stool [39]) and the majority (59%) had moderate parasite burdens (1,000–9,999 epg). Moderate parasite burdens are associated with the presence of relatively few adult parasites [40]. Clearly, our study did not have sufficient power to examine the effects of heavy parasite burdens with *T. trichiura* on faecal microbiota. However, other studies in experimental animals showed effects associated with high burdens of *Trichuris* on the microbiota of the colonic mucosa [15] or the luminal contents of the colon [14]. Pigs were challenged with 20,000 *T. suis* ova yielding an average of 3,222 adult worms [14] and the rhesus macaques with 1,000 *T. trichiura* ova [15]. In the case of *T. suis* infection of pigs, there are marked changes in the large intestinal mucosa at 21 days after infection, characterised by a catarrhal enteritis [12] with oedema coincident with the emergence of larvae at the mucosal surface. Similar changes might occur during experimental *T. trichiura* infections of rhesus macaque monkeys. In the case of chronic infections with *T. trichiura* in humans, the presence of parasites are not associated with marked histologic changes [6] except among symptomatic children with high parasite burdens (i.e. the *Trichuris* dysentery syndrome) [6]. One explanation, therefore, for why we were unable to replicate the findings of *Trichuris* studies done in experimental animals is that such changes that have been reported may reflect the inflammatory state of the intestinal mucosa.

Although the sample size of this study was relatively large in comparison to previous studies of human faecal microbiota, it is clear that we have sufficient power for detecting only relatively large perturbations in faecal microbiota and limited power for more subtle alterations that could still have significant immunological and metabolic effects in the human host. It should be acknowledged also that we cannot rule out the possibility that differences between our Ecuadorian cohort and individuals from the Western world might be influenced by methodological differences in sample storage and processing steps such as DNA extraction and PCR primer bias. It has been shown for example, that freezing samples can cause selected loss of *Bacteroides* from faecal samples [41],[42] and this might conceivably affect our monitoring of *Prevotella* to *Bacteroides* ratios. However, we did attempt to mitigate the effects of frozen storage by aliquoting faecal samples into 90% ethanol prior to freezing. The fact that we could detect up 8.9% *Bacteroides* in our samples also suggests that we did not suffer complete loss of *Bacteroides* spp. due to freezing. Furthermore, the apparent predominance of *Prevotella* spp. over *Bacteroides* spp. in other developing world cohorts [35],[36] suggests intriguing large-scale differences in microbiota composition between individuals from urban, industrialised societies and those from rural areas of less-developed regions, which is clearly worthy of further investigation.

In summary, we have investigated the effects of *T. trichiura* infections in children on the faecal microbiota. To our knowledge, this is the first study to investigate the effects of STH infections on intestinal microbiota in humans. We were unable to detect an effect of concurrent *T. trichiura* infections on the composition of the faecal microbiota compared to uninfected children, and treatment of *T trichiura* infections had no effect on the microbiota composition 21 days after treatment. Future studies should focus on the effects of *T. trichiura* infections in heavily infected children or in healthy unexposed volunteers and if possible, sample the intestinal contents or mucosa at the site of infection in the caecum. Because the effects of *T. trichiura* on intestinal microbiota may be persistent, such studies should collect samples at various time points after treatment and several control groups to control for past exposures may be appropriate. We also observed mixed infections with *A. lumbricoides* and *T. trichiura* to be associated with a reduced overall diversity of bacteria and particularly a reduction in the relative abundance of some members of the Clostridia Class. The significance of this is unclear and needs to be replicated in future studies.

## Supporting Information

Table S1 Barcode and primer details for each sample.
(XLSX)

Table S2 Frequency of the consumption of selected foods in a sample of 199 children aged 8 to 14 years from two of the study communities.
(DOCX)

Table S3 OTU distribution, and taxonomic classification across all samples.
(XLSX)

Table S4 Representative sequences (fasta format) for each OUT.
(XLSX)
