# Supplementary material for: Patent Human Infections with the Whipworm, Trichuris trichiura, Are Not Associated with Alterations in the Faecal Microbiota
Source: PLoS One. 2013 Oct 4;8(10):e76573. doi: 10.1371/journal.pone.0076573 (PMC3790696; doi:10.1371/journal.pone.0076573)
Supplement: Table S2 — Frequency of the consumption of selected foods in a sample of 199 children aged 8 to 14 years from two of the study communities. (DOCX) [file pone.0076573.s002.docx]

**Table S2:** Frequency of the consumption of selected foods in a sample of 199 children aged 8 to 14 years from two of the study communities.

| Food intake | Frequency of consumption | | |
| --- | --- | --- | --- |
|  | Never/occasionally | 1-4 times/week | Daily |
| Burgers  Other Meat  Fish  Shrimps  Fruit  Salad  Rice  Plantain  Yuca  Bread  Peanut  Potatoes  Eggs  Fizzy drinks | 15.6  6.0  5.5  49.8  32.1  24.4  0  0.5  57.3  3.6  85.4  6.5  5.0  19.1 | 39.2  86.9  55.3  44.7  48.7  62.9  3.0  5.0  38.2  28.7  11.6  46.2  43.7  51.2 | 45.2  7.1  39.2  5.5  28.1  12.7  97.0  95.0  4.5  67.7  3.0  47.3  51.3  29.7 |
